# A kinetic model for polyhydroxyalkanoates production by *Cupriavidus necator* using dark fermentation effluents

**DOI:** 10.1007/s10532-026-10377-9

**Published:** 2026-09-30

**Authors:** Ildefonso Caro, Agustín Romero-Vargas, Manuel Jesús Díaz, Julia Huertas

**Affiliations:** Department of Chemical Engineering and Food Technology, Faculty of Science, Universidad de Cádiz, Campus Río San Pedro, Puerto Real, 11510 Cádiz, Spain

**Keywords:** Polyhydroxyalkanoates, Volatile fatty acids, Fermentation kinetics, Dark fermentation effluents, Bio-refinery

## Abstract

Polyhydroxyalkanoates (PHAs) represent a family of biodegradable polymers that are precursors of bioplastics, offering a sustainable alternative to plastics derived from fossil fuels, within a circular economy framework. This study investigates the production of PHAs by the *Cupriavidus necator* bacterium, using as carbon sources synthetic media with volatile fatty acids (VFAs) and real effluents from dark fermentation (DFE) of seaweed wastes, which contain this type of acids. Experimental results indicate that, when acids are fermented singly, propionic acid yields a lower polymer quantity than acetic or butyric acids. However, when VFAs are supplied in mixtures, synergistic effects are observed. The kinetic models actually published fail to account for the increasing alkalinity of the medium during the VFA consumption, which frequently leads to biomass growth inhibition, and do not incorporate pH-dependent growth equations or consider the deleterious impact of alkaline conditions on the polymer stability. Thus, a novel kinetic model is proposed, based on the Monod equation, but integrating the inhibitory effects of pH in the biomass growth and the alkaline polymer degradation, with only nine fitting parameters. The new model allow for the robust prediction of the PHA production, the substrate consumption and the biomass growth, in the experimental conditions applied, which are typical of the PHA fermentations of DFE. The overall regression coefficient of theoretical curves (R^2^) is over 0.92, thus facilitating the designing and optimization of the industrial-scale processes.

## Introduction

Bioplastics have emerged as a pivotal sustainable alternative to conventional polymers derived from fossil-fuel precursors. Characterized either by their biodegradability or by their derivation from biological substrates, these materials facilitate the integration of plastic production into circular economy frameworks (Kourmentza et al. 2017; Khatami et al. 2021). Consequently, substantial scientific research is currently directed toward the development of industrial processes that ensure both the economic viability and environmental sustainability of these bioproducts. Polyhydroxyalkanoates (PHAs) serve as the primary chemical precursors for bioplastics. Their mechanical and physicochemical properties, including density, rigidity, and elasticity, are fundamentally dictated by their specific chemical structure (Sharma et al. 2021). Within the PHA family, poly-3-hydroxybutyrate (PHB), a polyester derived from 3-hydroxybutyric acid, is generally identified as the most relevant compound. Another prominent member is poly-3-hydroxyvalerate (PHV), the polyester derived from 3-hydroxyvaleric acid. Furthermore, various co-polymers composed of different beta-hydroxyacids are documented,  the most notably poly-(3-hydroxybutyrate-co-3-hydroxyvalerate) (PHBV). These compounds are categorized based on the length of their side chain into: short-chain-length (SCL), medium-chain-length (MCL) and long-chain-length (LCL) polymers, containing from 3 to 5 units, from 6 to 14 units or 15 units or more, respectively (Nigmatullin et al. 2015; Sun et al. 2007; Ramsay et al. 1991).

The production of these biopolymers can be primarily achieved through microbial fermentation, as numerous microorganisms possess the metabolic pathways necessary to convert diverse carbon substrates into 3-hydroxy acids, which are subsequently transformed into PHAs (Kim and Lenz 2001). These compounds function as intracellular energy reserves, mediators of stress resistance, redox balancing agents and survival resources, under fluctuating environmental conditions (Sedlaceka et al. 2019). They are usually produced when the culture medium exhibits a surplus of carbon source in combination with a deficiency in nitrogen or phosphorus source, critical nutrients for the primary growth metabolism (Lu et al. 2009). This metabolic accumulation can also be triggered by the depletion of other growth-limiting factors, such as the dissolved oxygen, the concentration of essential minerals (Davis et al. 2015; Annuar et al. 2008), or the redox stress (Cabecas-Segura et al. 2022). The diversity of microorganisms capable of PHA production is extensive, encompassing a large number of taxa (Wang et al. 2020). Additionally, complex natural cultures, such as those existing in sewage sludge and anaerobic fermenter microbiota, have demonstrated significant capacity for PHA formation (Vargas et al. 2014; Serafim et al. 2008, 2004; Saratale et al. 2021). In this microbiological landscape, the bacterium *Cupriavidus necator* stands out as one of the most prolific species for fermentative PHA production. This species has been the subject of extensive investigation due to its remarkable metabolic versatility, allowing for the assimilation of various carbon sources, including hexoses (glucose or fructose) (Horvat et al. 2013; Baei et al. 2011), polyols (glycerol or butadienol) (Spoljaric et al. 2013a; Fereidouni et al. 2011), alkanes (Raza et al. 2016) or volatile fatty acids (acetic, propionic, butyric, or valeric acid) (Cai et al. 2023). The literature further reports the successful fermentation of *C. necator* and related bacteria for PHA production using a wide range of complex natural substrates. These include some hydrolyzed lignocellulosic residues (Ahn et al. 2015; Muhammad et al. 2020), valued for their fermentable sugar content; non-edible oils (Arumugama et al. 2018), exploited because they do not compete with edible oils in the food industry; or dark fermentation effluents (DFE) (Mengmeng et al. 2009; Dionisi et al. 2009), which are utilized for their high volatile fatty acids (VFAs) concentration.

**Scheme. 1:**
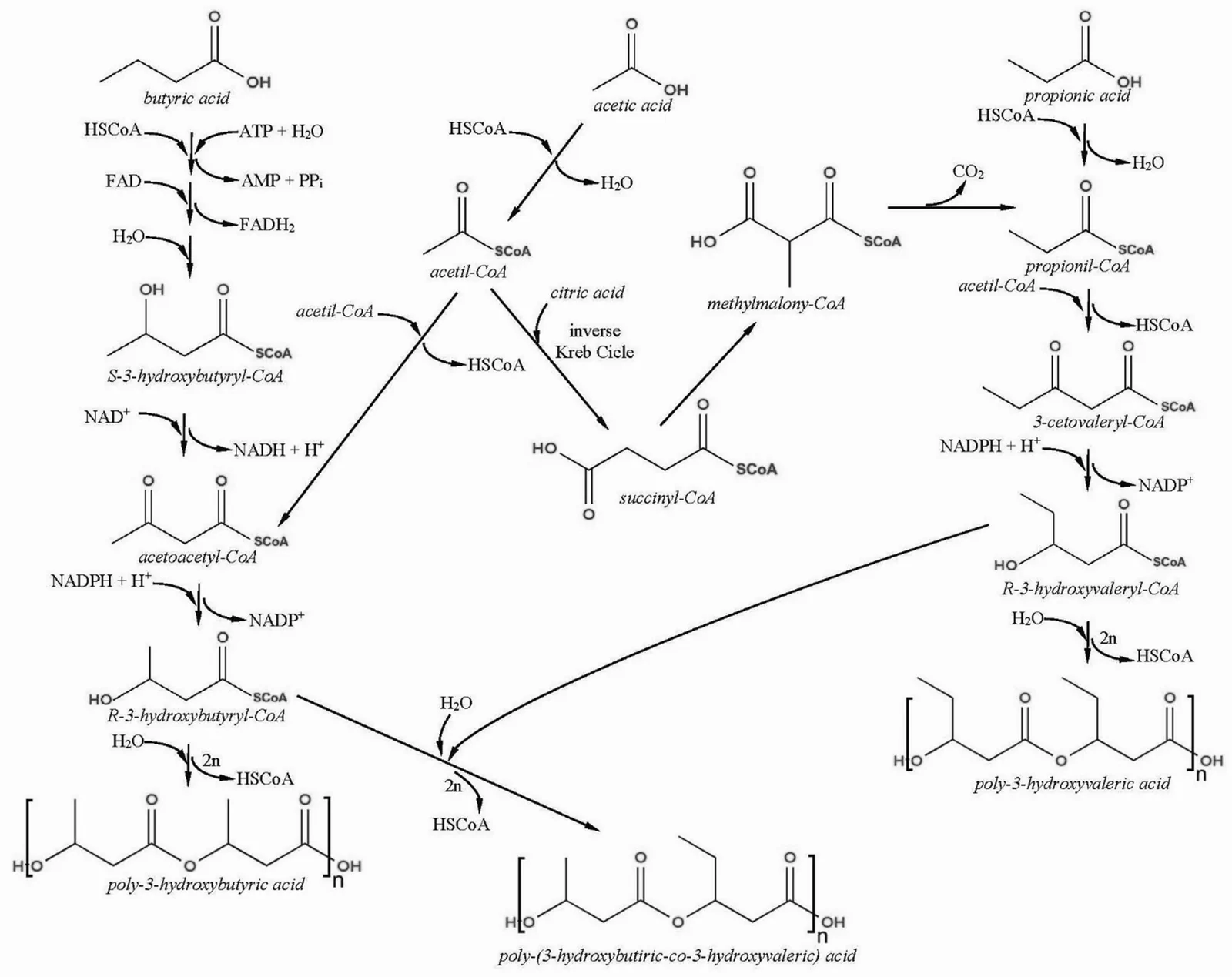
Resumed biochemical pathway of PHAs formation, from the three simplest VFAs.

Specifically, the utilization of dark fermentation effluents represents a particularly advantageous strategy for the industrial-scale production of PHAs, largely due to the reduction in process costs. Dark fermentation is a consolidated stage in the protocol of various wastewater and solid waste treatments (Gurieff and Lant 2007; Sekoai et al. 2021) and recently this process has been adapted to provide value-added applications for food industry by-products (Lin 2021), such as exhausted sugar beet pulp (Álvarez-Gallego et al. 2018; Gómez-Quiroga et al. 2019), and to mitigate environmental issues like coastal algal blooms (Fernández-Medina et al. 2022). The general composition of this last effluent includes: pH 4.5–6.0; total solids 15–80 g/L; DQO 10–40 g/L (mainly insoluble polysaccharide derivatives); ammoniacal nitrogen 1–2 g/L; VFAs 1–10 g(L; and tannins 1–3 g/L (Fernández-Medina et al. 2025). However, given the substantial variability in the physicochemical characteristics of the resulting effluents, the optimization of the *C. necator* fermentation remains a critical requirement to enhance the efficiency of the industrial production of PHAs. For instance, as illustrated by the metabolic pathways in Scheme 1, the assimilation of butyric acid (the C_4_ VFA) directly facilitates the output of the polymer poly-3-hydroxybutyrate (the C_4_ PHA). However, the conversion of propionic acid (the C_3_ VFA), results in the biosynthesis of poly-3-hydroxyvalerate (the C_5_ PHA) (Steinbüchel and Lütke-Eversloh 2003). This last biochemical process involves chain elongation through the addition of acetyl-CoA, derived from propionic acid via the decarboxylation of propionyl-CoA. The condensation of these precursors is primarily favored under elevated dissolved oxygen concentrations (Huong et al. 2024). Conversely, the assimilation of acetic acid (the C_2_ VFA) can facilitate the production of either PHB or PHV, depending on the intracellular oxygen balance. Thus, *C. necator* demonstrates a capacity for varying degrees of copolymerization modulated by oxygen availability (Ferre-Guell and Winterburn 2017; Salgaonkar and Bragança 2017), although PHB remains the predominant product under high aeration (Third et al. 2003). The establishment of accurate kinetic models is therefore essential for the quantitative prediction of product concentration and volumetric productivity in industrial processes.

Despite this biotechnological potential, few kinetic models have been published to date specifically addressing the fermentation of *C. necator* on VFAs. Initially, research by Wang et al. (Wang et al. 2010). proposed a Monod-type equation incorporating substrate inhibition, which was attributed to the acidogenic effects of high substrate concentrations. In studies utilizing several VFA proportions, characteristic of dark fermentation effluents, maximum specific growth rates between 0.24 and 0.40 h⁻^1^ were observed, with optimal total substrate concentrations identified between 10 and 12 g/L. Further advancements in kinetic modeling have addressed PHA production using caprylic acid (the C_8_ VFA) (Vega and Castillo 2018), providing a kinetic structure applicable to multiple species in both batch and continuous operating modes. However, these mathematical descriptions are frequently characterized by high complexity, often involving more than ten parameters. Similarly, models developed for propionic acid as the primary carbon source (Duvigneau et al. 2021) have been described as cumbersome, requiring over a dozen parameters. Due to this complexity, some studies have bypassed kinetic modeling entirely, opting for Response Surface Methodology (RSM) (Cai et al. 2023) to identify conditions of maximal productivity. Thus, comparative data suggest that acetic acid exhibits higher productivity relative to propionic, butyric, or valeric acids and empirical evidence indicates that an initial VFA concentration of approximately 10 g/L serves as the most productive threshold for substrate loading. While kinetic models exist for *C. necator* utilizing alternative carbon sources, such as carbohydrates, alcohols (Spoljaric et al. 2013b; Huong et al. 2017), or lignocellulosic hydrolysates (Yu and Si 2001), these mathematical structures often omit substrate inhibition terms and still maintain more than ten parameters. Consequently, the models derived from these substrates are fundamentally distinct and non-transferable to VFA-based fermentation systems like DFE. Furthermore, models tailored to microorganisms other than *C. necator* lack the specificity required for the optimization of processes based in DFE (Wang et al. 2020; Koller et al. 2006). Finally, extensive reviews have cataloged various PHA production models (Novak et al. 2015; Lhamo and Mahanty 2022), many of which are derived from experiments conducted at a neutral initial pH without active control, a strategy often favored in industrial settings to reduce operational costs. However, a significant limitation in these models is the failure to account for the increasing alkalinity of the medium during the VFA consumption, which frequently leads to biomass growth inhibition. To date, no published models incorporate pH-dependent growth equations or consider the deleterious impact of alkaline conditions on PHA polymer stability. Given the expanding role of dark fermentation in waste management, such as the mitigation of algal blooms (Fernández-Medina et al. 2022), there is a critical need for models that accurately integrate these pH-related phenomena. Consequently, this study proposes a novel kinetic model designed to account for these inhibitory effects and facilitate the optimization of PHA production by *C. necator* using VFA substrates.

## Material and methods

### Experimental design and process monitoring

The experimental protocol included a series of batch fermentation trials with VFAs as the primary carbon source (acetic acid, propionic acid and butyric acid), both solo and in mixes, and also included fermentation experiments with a pure standard carbohydrate (glucose) and experiments with a complex natural substrate (dark fermentation effluents, DFE). Glucose was applied as a reference substrate in order to be able to develop comparative efficiency analysis. The selection of the VFAs and their concentration ranges was strategically informed by their prevalence in dark fermentation effluents (Kumar et al. 2019), particularly those derived from the anaerobic treatment of the above mentioned algal solid wastes (Fernández-Medina et al. 2022; Romero-Vargas et al. 2023, 2024). Furthermore, single VFA experiments were prepared maintaining the initial acid concentration under the substrate inhibition range (around 8 g/L) and the VFA mixtures were prepared trying to reproduce the ratios of the genuine dark fermentation effluents (acetic-propionic-butyric, 12–1–2). The initial concentrations of each compound applied in each experiment are shown in Table 1.

**Table 1:** Initial molar concentration of each carbon source (mM) in the experiments

EXPERIMENT^*^	glucose	Acetic acid	Propionic acid	Butyric acid
GLU	42	–	–	–
ACE	–	142	–	–
PRO	–	–	154	–
BUT	–	–	–	90
2AB	–	100	–	11
3HC	–	149	10	17
3LC	–	15	1.2	1.6
DFE	–	23	3	2.6
*M*_*w*_	180.063	60.052	74.079	88.106
*pK*_*a*_	–	4.756	4.880	4.820

^*^Carbon source in each experiment: *GLU* glucose; *ACE* acetic acid; *PRO* propionic acid; *BUT* butyric acid; *2AB* binary mixture of acetic and butyric acid; *3HC* ternary mixture of acids at high concentration; *3LC* ternary mixture of acids at low concentration; *DFE* dark fermentation effluent.

Molecular weight (Mw) in g/mol and acid dissociation constant (pKa)

Furthermore, in the experiments with glucose as the substrate, solid glucose was added to the medium at the sixth day (until reaching 50 mM), applied in a fed-batch mode as it is usually done to avoid substrate inhibitions. The re-addition was done directly when the stabilization of the biomass growth was observed, due to this kinetic variable was able to be easily determined daily. The aim of this test was to analyze the capacity of the microbial metabolism to recover the biomass growth and the PHA biosynthesis. Finally, to accurately delineate the kinetic behavior of the system, systematic monitoring was conducted in each experiment for three key variables: substrate depletion (VFAs), total biomass accumulation (whole cells), and product formation (PHAs). All the experiments were performed in triplicate and the data reported in Figures are the mean values with the Student’s t confidence intervals (95% probability).

### Microbial strain and inoculation protocol

The biological agent utilized in this study was the strain *Cupriavidus necator* DSM 545, obtained from the Spanish Collection of Type Cultures (CECT 4635). This bacterium was selected for its high metabolic proficiency in synthesizing PHAs from VFAs. For long-term preservation, the supplied strain ampoule was re-suspended in 100 mL of a glycerinated aqueous solution (50:50 in weight), and maintained at −70 ºC until use. The inoculation protocol was standardized to ensure physiological consistency. Thus, 1 mL of the cryopreserved suspension was revived in 20 mL of a sterilized nutritive medium and incubated at 30 ºC and 250 rpm, during approximately 24 h, until an optical density between 4.0 and 5.0 AU (600 nm) was reached. This culture constituted the inoculation culture. The sterilized nutritive medium comprised 5 g/L meat extract, 10 g/L peptone, 5 g/L NaCl and was adjusted to pH 7.2 with a 0.1 M NaOH solution. Finally, inoculation of the production flasks was performed adding a 5% (v/v) of the inoculation culture to the fermentation medium in each case.

### Fermentation conditions and media composition

All fermentation processes were conducted in 500 mL Erlenmeyer flasks with a working volume of 100 mL, incubated at 30 ºC under constant agitation at 250 rpm. The basal fermentation medium was formulated with the following macronutrients concentrations to ensure their ample availability: (NH_4_)_2_SO_4_ (1 g/L), MgSO_4_·7H_2_O (0.2 g/L), KH_2_PO_4_ (1.5 g/L), and Na_2_HPO_4_·H_2_O (9 g/L). This medium was supplemented with 1 mL/L of the following trace element solution to guarantee their presence: FeSO_4_·7H_2_O (10 g/L), ZnSO_4_·7H_2_O (2.25 g/L), CuSO_4_·5H_2_O (1 g/L), MnSO_4_·H_2_O (0.5 g/L), CaCl_2_·2H_2_O (2 g/L), Na_2_B_4_O_7_·7H_2_O (0.23 g/L), Na_2_MoO_4_·2H_2_O (0.1 g/L) and 10 mL/L of an aqueous solution of HCl at 13 % v/v. The initial pH of all fermentation media was adjusted before sterilization to 7.2, using a 0.1 M NaOH solution.

Some of the PHA fermentation experiments were carried out using dark fermentation effluents as the substrate. These effluents were obtained from other dark fermentation studies in our laboratory and all the effluent features are detailed in bibliography (Fernández-Medina et al. 2025; Rouabhia et al. 2026). The raw liquid was initially processed via fiberglass filtration to remove the suspended solids. This filtered effluent was subsequently supplemented with the aforementioned mineral salts and trace elements, and pH was adjusted to 7.2 using a 0.1 M NaOH solution, before sterilization, in order to achieve conditional parity with the synthetic media.

### Sampling procedure and analytics

Process monitoring was conducted via the systematic collection of dual 1.0 mL aliquots under aseptic conditions throughout the fermentation cycle. The primary aliquot was dedicated to the quantification of biomass density and residual substrate concentrations, while the secondary aliquot was processed for polyhydroxyalkanoate determination. Biomass concentration was assessed spectrophotometrically at 600 nm and subsequently correlated to cell dry weight (g-DW/L) through a prevalidated calibration curve (Slowik et al. 2023). To ensure sample integrity for subsequent glucose or VFA analysis, the aliquots were centrifuged at 5,000 g, filtered through 0.22 μm membranes and stored at −20 ºC until further use. Glucose quantification was achieved using the 3,5-dinitrosalicylic acid colorimetric method for reducing sugars (DNS), optimized for 96-well microplate high-throughput analysis (Gonçalves et al. 2010). Since glucose was the only carbohydrate within the medium, this method ensured high specificity for its determination. The determination of VFA profiles was executed using gas chromatography (GC) equipped with FID and a Nukol™ capillary column. Hydrogen served as the carrier gas, and phenol (600 ppm) was integrated as an internal standard. Calibration lines were created using standards of each pure VFA (Gómez-Quiroga et al. 2019).

The aliquot samples for PHA determination were stored at −20 ºC after adding 0.2 mL of aqueous solution of sodium hypochlorite (10 % v/v), in order to stop the fermentation process. For the quantification of intracellular PHAs, the polymers were subjected to acid-catalyzed methanolysis to generate their respective methyl ester derivatives (Crognale et al. 2022). This depolymerization process involved treating the cellular pellet of each sample with 2 mL of acidic methanol (3 % v/v H_2_SO_4_) and 1 mL of chloroform (containing 100 ppm of heptadecane as an internal standard), and heating the suspension at 100 °C for 4 h within hermetically sealed vessels. Following phase separation, obtained after adding 1 mL of distilled water and cooling to 4 °C overnight, the methyl esters were analyzed in the aqueous phase via GC-FID utilizing a Chrompack™ CPSIL-5CB capillary column. Nitrogen was used as the carrier gas and the chromatographic thermal program was the following: injector at 250 ºC and oven 5 min at 80 ºC, 5 min rising at 8 ºC/min, 1 min at 120 ºC, 2 min rising at 50 ºC/min, and 5 min at 220 ºC. Calibration lines were generated by full processing different weights of a commercial poly(3-hydroxybutyrate-co-3-hydroxyvalerate) standard 8 % mol PHV (Sigma-Aldrich®, Germany) (Braunegg et al. 1978).

### Kinetic model

Consistent with established postulates for kinetic fermentation of polyhydroxyalkanoates, the total microbial biomass (*X*) is mathematically partitioned into two distinct fractions: the accumulated bioplastic product (*P*) and the rest of the residual cell biomass (*R*) (Annuar et al. 2008; Vega and Castillo 2018; Somashekara et al. 2009). Thus, the following fundamental mass balance in terms of concentrations is defined:

1 X=R+P

The residual fraction *R* is identified as the component of the active biomass that is endowed with the capacity for self-multiplication. Thus, the biomass growth is based only on the intervention of the *R* part, according to a Monod-type equation with the Edwards modification for substrate inhibition:

2 $$\left. {\frac{dR}{{dt}}} \right|_{growth} = \mu_{R} R$$

3 $$\mu_{R} = \mu_{R}^{o} \frac{S}{{K_{R} + S}} \cdot e^{{ - \frac{S}{{K_{I} }}}}$$

Here, *μ*_*R*_ is the specific growth rate of biomass, referred to the residual part R, ${\mu}_{R}^{o}$ is the maximum specific growth rate, *K*_*R*_ is the Monod saturation constant for growth, *K*_*I*_ is the substrate inhibition constant, and *S* is the substrate concentration (in this case, the volatile fatty acids). In this model, the Edwards modification has been introduced for substrate inhibition rather than other alternatives (for example, Haldane). The mathematical expression of the Edwards’ equation drives to a more severe asymptotic curve and we think this better represents the drastic influence of acidic compounds that is generally observed on microbial growth. Moreover, the Haldane’s equation bases the inhibition on a mechanism of reversible saturation of metabolic routes (non-competitive substrate inhibition), while Edwards’ equation supposes an irreversible degradation of the growth processes, as is usually produced by acids. Actually, as we have mentioned above, all the metabolic activity of *C. necator* is highly sensitive to the medium acidity, with the optimal pH for growth identified at 6.8 ± 0.4 (Arumugama et al. 2018; Trigueros et al. 2017).

Although these culture media are typically fitted to an initial pH of 7.2, the system exhibits significant alkalization as the process advances (Steinbüchel and Lütke-Eversloh 2003). This phenomenon is primarily attributed to the metabolic assimilation of VFAs, which leads to the stoichiometric liberation of hydroxyl anions (OH^–^) from the alkali previously added for the pH stabilization. To quantify this acidic shift, the molar concentration of the hydroxyl anion (*OH*) is here modeled as a direct function of the cumulative acid consumption. Thus, utilizing the theoretical material balance for a salt solution formed from a weak acid (VFAs) and a strong base (NaOH), the change in hydroxyl concentration (*ΔOH*) is calculated applying the acid dissociation constant (*K*_*a*_) and the ionic product of water (*K*_*w*_). This allows for the precise determination of pH evolution during the fermentation cycle based on the consumption of acid, which can be determined from the difference between the instantaneous substrate concentration (*S*) and the initial substrate loading (*S*_*o*_).

4 $$\Delta OH = \frac{{K_{w} }}{\Delta H}$$

5 $$\Delta H = \sqrt {\frac{{K_{w} K_{a} }}{{\left( {S_{o} - S} \right) + K_{a} }}}$$

6 $$pK_{w} = pH + pOH = pH_{o} + pOH_{o} = 14$$

7 $$pOH_{o} = 14 - pH_{o} = 14 - 7.2 = 6.8$$

8 $$OH = OH_{o} + \Delta OH = 10^{ - 6.8} + \Delta OH = 10^{ - 6.8} + \frac{{K_{w} }}{{\sqrt {\frac{{K_{w} K_{a} }}{{\left( {S_{o} - S} \right) + K_{a} }}} }}$$

9 $$pH = 14 - pOH = 7.2 + \log \frac{{K_{w} }}{{\sqrt {\frac{{K_{w} K_{a} }}{{\left( {S_{o} - S} \right) + K_{a} }}} }}$$

Due to the substantial substrate concentrations often initially applied (around 0.1 M), the medium typically reaches a terminal pH of approximately 9.0. At this threshold, the elevated concentration of OH^–^ anions becomes sufficient to induce biomass degradation. Then, the specific rate of biomass degradation (*μ*_*E*_) is proposed to be related to the specific rate of the bioplastic product degradation (*μ*_*D*_), since both phenomena are considered a consequence of the pH evolution, as will be seen later. Thus, the first is directly linked to the second by the proportionality constant (${\mu}_{E}^{o}$):

10 $$\left. { - \frac{dR}{{dt}}} \right|_{degradation} = \mu_{E} R$$

11 $$\mu_{E} = \mu_{E}^{o} \mu_{D}$$

The resulting evolution of the residual part of biomass is therefore characterized by the superposition of two competing phenomena: growth and degradation. Initially, biomass proliferation dominates in the system, facilitated by high substrate availability and near-optimal pH levels. Subsequently, as substrate depletion occurs and alkalinity increases toward pH 9.0, the biomass decay prevails. This model accounts for the dynamic transition between the growth and degradation phases as a function of the chemical environment. Thus, the complete kinetic behavior of the residual biomass is described by the following differential equation:

12 $$\frac{dR}{dt}={\left.\frac{dR}{dt}\right|}_{growth}+{\left.\frac{dR}{dt}\right|}_{degradation}=\left({\mu}_{R}-{\mu}_{E}\right) R$$

13 $$\frac{dR}{dt}=\left({\mu}_{R}^{o} \frac{S}{{K}_{R}+S}\cdot {e}^{- \frac{S}{{K}_{I}}}-{\mu}_{E}^{o} {\mu}_{D}\right) R$$

Regarding the product (*P*), the polyhydroxyalkanoate polymer, the biosynthesis is also described by a Monod-type expression, similar to that used for the biomass growth, as the production metabolism is also considered inhibited by the presence of acids. As a result, the specific rate of product formation (*μ*_*P*_) is modulated by the maximum specific production rate (${\mu}_{P}^{o}$), the Monod saturation constant (*K*_*P*_) and the inhibition constant (*K*_*I*_), which is considered to be the same as for growth inhibition. Then, the biopolymer accumulation is significantly attenuated at elevated acid concentrations, following the next equation:

14 $$\left. {\frac{dP}{{dt}}} \right|_{formation} = \mu_{P} R$$

15 $$\mu_{P} = \mu_{P}^{o} \frac{S}{{K_{P} + S}} \cdot e^{{ - \frac{S}{{K_{I} }}}}$$

**Scheme. 2:**
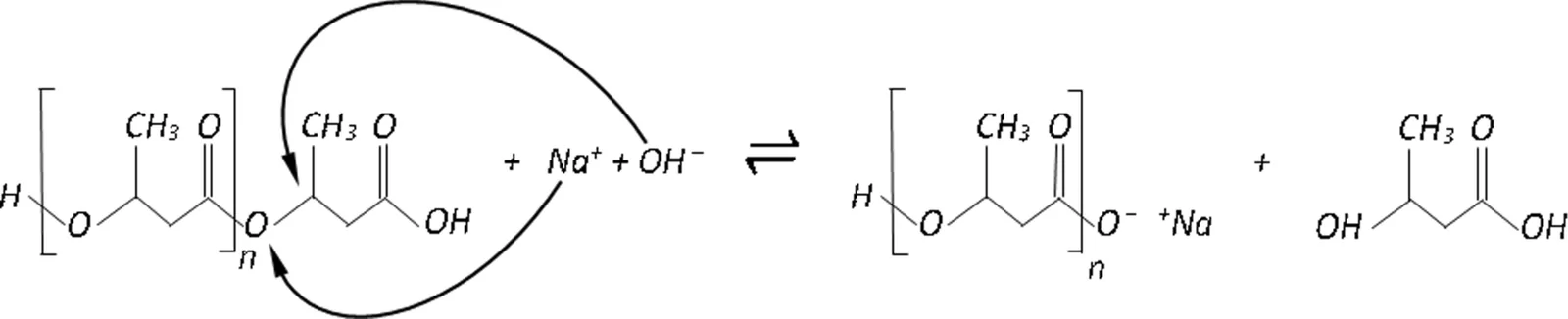
Reversible saponification reaction of the polyhidroxybutirate polymer (PHB), occurring by nucleophilic attack of the ester bond.

On the other hand, the structural stability of the synthesized PHA, which is actually a polyester, is compromised by the alkaline medium, which facilitates saponification. This chemical decomposition is particularly evident in polymers such as polyhydroxybutyrate (PHB) when exposed to high concentrations of hydroxyl anions. As biomass degrades in high pH media, it can also be considered that PHA glomeruli are exposed to the pH attack in such conditions and are then degraded. In summary, the polymer degradations is modeled as a second-order reversible reaction (Scheme 2), where the net rate of polymer decomposition is a function of four concentrations: the product that is degraded (*P*), the hydroxyl anions (*OH*), the monomer resulting from degradation (*D*) and the polymer part that remains after degradation (P^*^). The kinetic constants *k*_*1*_ and *k*_*2*_ represent respectively the forward and reverse rate constants of the reaction:

16 $$P + OH \begin{array}{*{20}c} {k_{1} } \\ \rightleftharpoons \\ {k_{2} } \\ \end{array} P^{*} + D$$

17 $$\left. { - \frac{dP}{{dt}}} \right|_{decomposition} = k_{1} \cdot P \cdot OH - k_{2} \cdot P^{*} \cdot D$$

Since P^*^ and *P* represent nearly the same molar concentration at any instant, we can use the following decomposition equations:

18 $$\left. { - \frac{dP}{{dt}}} \right|_{decomposition} = k_{1} \ \ P \ \ OH - k_{2} \ \ P^{*} \ \ D = \mu_{D} \ P$$

19 $$\mu_{D} = k_{1} OH - k_{2} D$$

where *μ*_*D*_ is the above mentioned specific rate of PHA decomposition. Due to the stored polymer in cells can also be biochemically metabolized by intracellular PHA-depolymerases, in order to recover biomass viability once the external substrate is depleted, this parameter includes simultaneously both stages of degradation (chemical and biochemical). Thus, the net concentration of PHA (*P*) throughout the fermentation cycle is determined by the differential equilibrium between metabolic formation and decomposition.

20 $$\frac{dP}{{dt}} = \left. {\frac{dP}{{dt}}} \right|_{formation} + \left. {\frac{dP}{{dt}}} \right|_{decomposition} = \mu_{P} R - \mu_{D} P$$

Consequently, the resultant kinetic profile of PHA typically exhibits an initial phase of accumulation followed by a subsequent declination, as the medium increasingly becomes alkaline. The specific evolution of D monomers in the media (cells and solution) was not the focus of this study and was not determined, but their formation rate must be stoichiometrically coupled to the rate of polymer degradation.

21 $$\frac{dD}{{dt}} = \left. { - \frac{dP}{{dt}}} \right|_{decomposition} = \left( {k_{1} OH - k_{2} D} \right) P = \mu_{D} P$$

Finally, substrate consumption is assumed to be governed by growth-associated kinetics, relative to the total biomass, which accounts for both the growth of the residual fraction of cells and the accumulation of biopolymer.

22 $$- \frac{dS}{{dt}} = Y_{SX} \frac{dX}{{dt}} = Y_{SX} \left( {\frac{dR}{{dt}} + \frac{dP}{{dt}}} \right)$$

To facilitate the application of the model to industrial optimization tasks, the mathematical framework has been defined by trying to reduce the number of the fitting parameters involved. Thus, only nine coefficients have been finally included: ${\mu}_{R}^{o}$, *K*_*R*_, ${\mu}_{P}^{o}$, *K*_*P*_, ${\mu}_{E}^{o}$,* K*_*I*_, *k*_*1*_, *k*_*2*_, and* Y*_*SX*_. This approach allows for the accurate simulation of the process without using redundant parameters, and the evolution of the three kinetic curves (substrate, biomass and product) is calculated taking on only three coefficients per each. The supplemental constants included in the model, such as molecular weights or acid dissociation constants, were directly taken from the existing literature (Table 1). The integration of the system of differential equations of the model has been carried out numerically, applying the Runge–Kutta 4^th^ order algorithm (ode45 function of MATLAB^®^) and introducing the experimental initial concentrations as limiting conditions. The initial set of parameters introduced in each fitting were the general average value observed in literature for similar kinetic models. The optimization of the nine fitting kinetic parameters was performed via iterative nonlinear regression, utilizing the damped least squares algorithm (Marquardt 1963). This rigorous numerical procedure aims to minimize the residual sum of squares between the *n* experimental observations (*exp*_*i*_) and the corresponding theoretical predictions (*teo*_*i*_) for all variables. The robustness of the fit was validated by maximizing the nonlinear regression coefficient (*R*^*2*^).

23 $$R^{2} = 1 - \frac{{\mathop \sum \nolimits_{i = 1}^{n} err_{i}^{2} }}{{\mathop \sum \nolimits_{i = 1}^{n} des_{i}^{2} }}$$

24 $$err_{i}^{2} = \left( {\exp_{i} - teo_{i} } \right)^{2} \hspace{2 cm} des_{i}^{2} = \left( {\exp_{i} - ave} \right)^{2} \hspace{2 cm} ave = \frac{{\mathop \sum \nolimits_{i = 1}^{n} \exp_{i} }}{n}$$

## Results and discussion

The set of experimental conditions of this study includes discrete carbon sources (glucose, acetic acid, propionic acid and butyric acid) and multi-component mixtures of VFAs (binary and ternary). These last trials were carried out to identify potential synergistic interactions between organic acids and to reproduce the real industrial-scale dynamics. Moreover, as mentioned above, dark fermentation effluents were also tested, which were derived from the anaerobic treatment of algal wastes in other studies.

### Single carbon sources

**Fig. 1:**
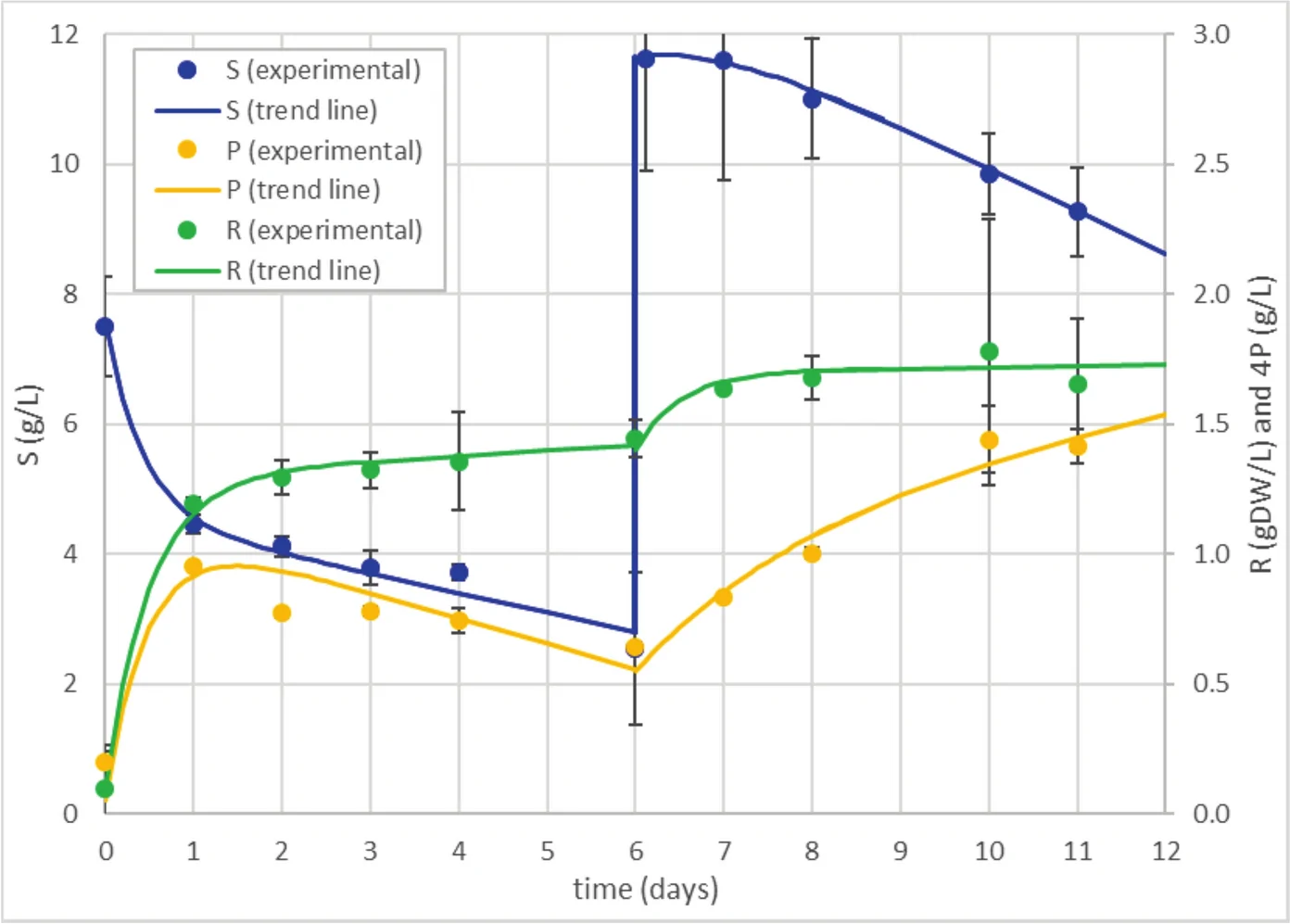
Fermentation evolution when glucose is the only carbon source (re-addition at sixth day). Concentration of substrate (*S*), residual biomass (*R*) and product (*P*). Average data and confidence intervals for t-Student probability of 95 %. Lines represent only tendencies. *P* is plotted at four times its original value (*4P* scale)

In the experiments utilizing glucose as the primary carbon source (Fig. 1), substrate depletion is shown to be growth-associated, with biomass accumulation following an asymptotic trajectory toward a saturation threshold on the sixth day. In relation to the PHA formation, although product accumulation was initially observed, a degradation phase entered subsequently as it is commonly observed in this type of fermentations (Horvat et al. 2013). Thus, during the first 24 h, around 6.2 % (w/w) of the consumed substrate is converted into biopolymer. However, this is later degraded progressively and the overall conversion ratio drops to near 2.2 %. After glucose re-addition, the ratio is recovered and achieves again the value of 6.3 %, along the next 6 days. In this second phase, the observed biomass growth is very short, so cells would supposedly be in a pure bioproduction stage. These low conversion ratios seem to indicate that, under these conditions, the PHA biosynthesis by this strain from glucose is less efficient than from VFAs (around fourfold lower, as it will be seen later). As glucose metabolism is different from that of the VFAs (Steinbüchel and Lütke-Eversloh 2003), involving the previous biosynthesis of the organic acids from glucose, the PHA production could be less efficient in strains not well adapted to this carbon source. Moreover, the VFA consumption includes the release of hydroxyl anions and the alkaline-mediated degradation, which are not present in the glucose fermentations. Consequently, the kinetic model proposed here could not be applicable to glucose-based systems. It is important to notice that lines drawn in Fig. 1 are data trends and they are plotted only to facilitate the curves tracking. In any case, fermentative sources containing glucose (as hydrolyzed agro-food residues) are in general more expensive than those containing VFAs (as DFE of seaweed) and this limits its industrial use for PHA production.

**Fig. 2:**
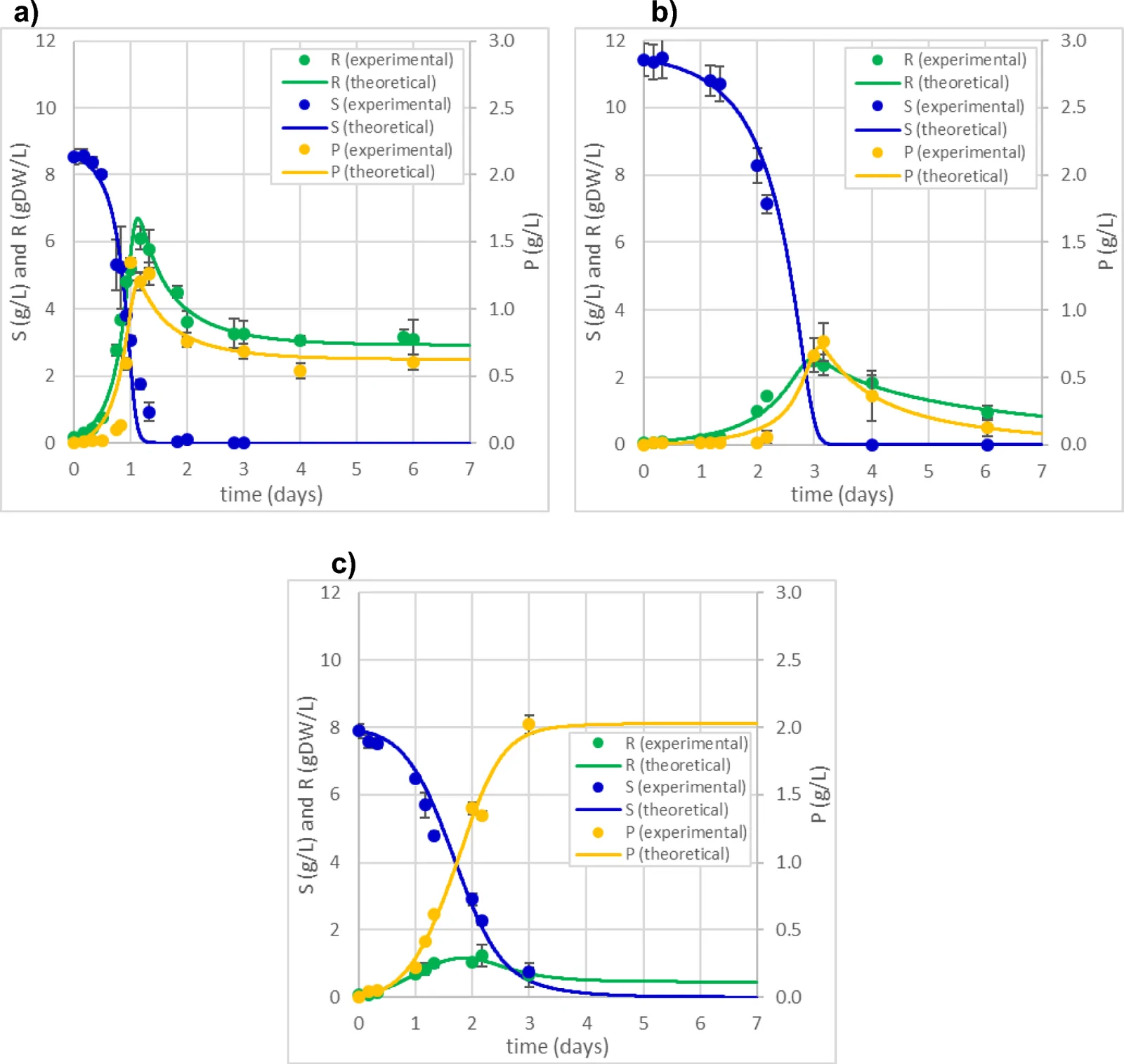
Fermentation evolution when a single volatile fatty acid is the carbon source: **a** acetic acid; **b** propionic acid; **c** butyric acid. Concentration of substrate (*S*), residual biomass (*R*), and product (*P*). Confidence intervals for t-Student probability of 95 %

Regarding the fermentations conducted with individual VFAs as the primary carbon source (Fig. 2), they exhibit a characteristic inverse S-shape of substrate depletion, accompanied by a transient expansion and subsequent decay of microbial biomass, after substrate exhaustion. In the case of the product (PHAs), its evolution followed a similar trajectory to biomass (accumulation and depletion). Notably, propionic acid demonstrated significantly lower PHA productivity compared to acetic or butyric acids, in accordance with other published works (Cai et al. 2023).

### Model fitting and evaluation

#### Single VFAs

The proposed model was independently fitted to the experimental data for each volatile fatty acid and the obtained parameters are shown in Table 2. The results demonstrated reasonable consistency across the trials, achieving an average regression coefficient around 0.96, which can be considered acceptable for microbial fermentation experiments. Besides, the initial pHs that have been set in the media are limiting conditions of the mathematical model (7.2), and the calculated final pHs match well in all cases with the experimental value obtained (around 9.0), with similar precision than regression coefficients. Regarding the growth parameters, the maximum specific growth rates (${\mu}_{R}^{o}$) appear to exhibit in this case values far above the reported in other published models for *C. necator*, being here around 2.666 g-R/g-R·h, with a relative mean deviation between substrates of 4 %. However, given the type of inhibition equation introduced in this model, their values are not actually achievable in practice; this is merely a mathematical coefficient that must be analyzed in combination with *K*_*R*_ and *K*_*I*_. In the case of the saturation constant for growth (*K*_*R*_), the average value is 44.7 g-S/L (0.60 M), with acetic acid recording the lowest values, thereby confirming the superiority of this substrate for the cellular proliferation of the strain. Finally, the substrate inhibition constant (*K*_*I*_) was identified as around 6.0 g-S/L (0.08 M), with also very low deviations between substrates (9 %). This suggests that the growth metabolism of *C. necator* is very sensitive to inhibition by VFAs, even at low titers.

**Table 2:** Model fitting parameters for each single volatile fatty acid

parameter	units	acetic	propionic	butyric	x̄	RMD
${\mu}_{R}^{o}$	g-R/g-R·h	2.817	2.660	2.523	2.666	4
*K*_*R*_	g-S/L	22.5	76.9	34.8	44.7	48
*K*_*I*_	g-S/L	5.2	6.2	6.6	6.0	9
${\mu}_{P}^{o}$	g-P/g-R·h	0.954	0.052	0.163	0.389	97
*K*_*P*_	g-S/L	49.6	0.6	2.6	17.6	121
*Y*_*SX*_	g-S/g-X	0.761	2.387	0.841	1.330	53
${\mu}_{E}^{o}$	-	1.281	0.486	11.161	4.309	106
*k*_*1*_	L/mol-OH·s	2.017	1.597	0.825	1.480	29
*k*_*2*_	·10^–5^ L/g-D·s	1.835	1.110	0.859	1.268	30
*R*^*2*^	–	0.940	0.981	0.982	0.968	2
*S*_*Rm*_	g-S/L	4.36	5.79	5.71	5.29	15
*μ*_*Rm*_	g-R/g-R·h	0.198	0.074	0.151	0.141	32
*S*_*Pm*_	g-S/L	4.76	1.68	3.05	3.16	34
*μ*_*Pm*_	g-P/g-R·h	0.034	0.029	0.056	0.039	28

x̄: average value; *RMD* relative mean deviation (%); *R*^*2*^ regression coefficient: *S*_*Rm*_ substrate concentration for the maximal specific growth rate; *μ*_*Rm*_ maximal specific growth rate; *S*_*Pm*_ substrate concentration for the maximal specific production rate, *μ*_*Pm*_ maximal specific production rate.

As can be observed, the growth-substrate curve (Eq. 3) present a maximum of *μ*_*R*_ that can be calculated applying the mathematical maximum condition. Thus, the maximal value of the specific growth rate that is truly achievable in practice (*μ*_*Rm*_), and the value of the substrate concentration that produce this rate (*S*_*Rm*_), are the following:

25 $$S_{Rm} = \sqrt {\left( {\frac{{K_{R} }}{2}} \right)^{2} + K_{R} K_{I} } - \frac{{K_{R} }}{2}$$

26 $$\mu_{Rm} = \mu_{R}^{o} \frac{{S_{Rm} }}{{K_{R} + S_{Rm} }} \cdot e^{{ - \frac{{S_{Rm} }}{{K_{I} }}}}$$

Both values can be obtained by simply combining the three growth parameters in each case (${\mu}_{R}^{o}$, *K*_*R*_ and *K*_*I*_). The results are also shown in Table 2. These values confirm the reported data for the range of growth of *C. necator* on VFAs (0.1–0.2 h^−1^)). Moreover, they are consistent with the generally recommended value for the initial VFA concentration (10 g/L).

Regarding the production parameters, the maximum specific production rate (${\mu}_{P}^{o}$) averaged 0.389 g-P/g-R·h, considerably below the one for the specific growth rate and evidencing the metabolic delay of production over growth. In this case, the relative mean deviation between substrates is high (97 %) and propionic acid shows a significantly lower value than the other acids, which places this substrate as a deficient source for PHA production. The saturation constant for production (*K*_*P*_) averaged a value of 17.6 g-S/L (0.29 M) with also very high variability between substrates (121 %), showing again that the production curve is different for each acid.

Analogously to the growth-substrate curve, the production-substrate curve (Eq. 15) present a maximum of *μ*_*P*_ that can also be calculated by simply combining the three production parameters in each case (${\mu}_{P}^{o}$, *K*_*P*_ and *K*_*I*_). Thus, the maximal value of the specific production rate (*μ*_*Pm*_), and the value of the substrate concentration that produce this rate (*S*_*Pm*_), are the following:

27 $$S_{Pm} = \sqrt {\left( {\frac{{K_{P} }}{2}} \right)^{2} + K_{P} K_{I} } - \frac{{K_{P} }}{2}$$

28 $$\mu_{Pm} = \mu_{P}^{o} \frac{{S_{Pm} }}{{K_{P} + S_{Pm} }} \cdot e^{{ - \frac{{S_{Pm} }}{{K_{I} }}}}$$

These results are also shown in Table 2. Again, the maximal rates can be theoretically achieved for very low substrate concentrations. Then, it is clear that the maximal efficiency of the process would be obtained at substrate concentrations under 5 g-S/L.

In relation to the rest of kinetic parameters, the yield coefficient of substrate to biomass (*Y*_*SX*_) has an average value of 1.330 g-S/g-X, with certain variability between substrates (53 %), showing that metabolic yield varies by acid. Thus, propionic acid demonstrated lower efficiency than the others, likely due to less oxygen and water assimilation in a more anoxic metabolism. Additionally, the proportionality constant for biomass degradation (${\mu}_{E}^{o}$) achieves an average value of around 4.309 (dimensionless), although a very high variability between acids is observed (106 %). Therefore, butyric acid seems to be much more effective at degrading the cell structure of this strain than the other acids (${\mu}_{E}^{o}$ around 10 times higher).

While it has been observed that growth and production parameters vary regularly with the type of acid, the values of kinetic constants for the PHA depolymerization remain more homogeneous across trials (variabilities between substrates of only 29 and 30 % respectively). Likely, because the nature of this process (a chemical and/or biochemical stage) is simpler than that of the metabolic process of growth or production. The direct rate constant (*k*_*1*_) was measured on an average value of 1.480 L/mol-OH·s and the reverse constant (*k*_*2*_) on 1.268·10^–5^ L/g-D·s. The direct constant significantly outweighs the reverse one, although it should be noted that they refer to different units. However, even if the second one were also expressed in molar terms, it would have a significantly lower value (the molecular weight of D should be between 50 and 100 g-D/mol-D and so *k*_*2*_ would be in the order of 10^–3^ L/mol-D·s). Therefore, these results indicate that the thermodynamic equilibrium of the mentioned reaction is shifted to the right, and the PHA polymers maintains certain stability under the specific environment of these fermentations.

#### Mixed VFAs

**Fig. 3:**
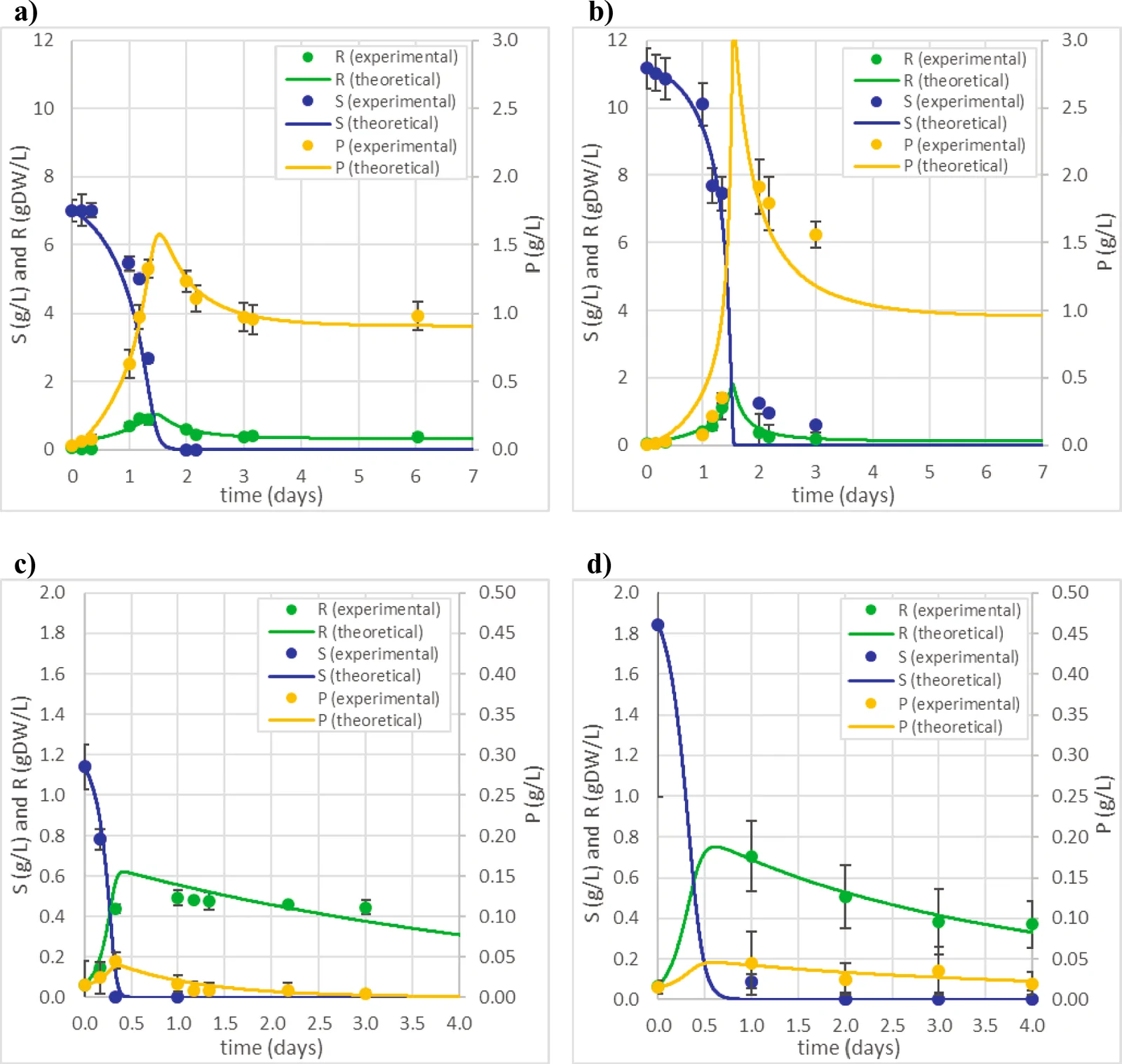
Fermentation evolution when using mixtures of volatile fatty acids as the carbon source: **a** 2AB experiment; **b** 3HC experiment; **c** 3LC experiment; **d** DFE experiment. Concentration of substrate (*S*), residual biomass (*R*), and product (*P*). Confidence intervals for t-Student probability of 95 %

Experimental data concerning fermentation with synthetic mixtures of VFAs **(**Figs. 3a, b and c) indicate that kinetic profiles are congruent with those observed for single substrates. Specifically, it can be noted that the experiment with low initial carbon load (Fig. 3c) exhibits faster substrate depletion and accelerated dynamics (note that both axes of this trial are scaled significantly shorter). For the mathematical fitting of parameters, the values were first prorated according to each acid’s ratio in the mixture and, later, they were optimized as in the single acid trials. The relative deviations between the fitted and prorated values were also computed. Results are shown in Table 3. As can be seen, some parameters maintain their values relatively near the prorated value, but others change significantly, which indicates that there must be synergistic effects between VFAs. These types of effects could be explained by considering that, since some parts of the VFAs metabolism are common, it is not necessary to duplicate the involved metabolites when several acids are consumed simultaneously. Therefore, the general metabolism would be more efficient, and the kinetic parameters would not correspond to the direct prorating of the individual parameters. It has also been observed that kinetic evolutions do not exhibit patterns of diauxic consumption (successive S-shaped curves), which supports the hypothesis that *C. necator* metabolizes all present VFAs simultaneously. On a different note, it has been observed that the regression coefficients obtained now are slightly lower than the ones obtained before, probably due to the higher complexity of the phenomena been modelled and the attempt to maintain few parameters in the model.

**Table 3:** Fitting parameters for the experiments with synthetic mixtures of VFAs and real DFE

		Experiment							
		2AB		3HC		3LC		DFE	
parameter	units	pro	fit	pro	fit	pro	fit	pro	fit
${\mu}_{R}^{o}$	g-R/g-R·h	2.806	3.233	2.797	5.455	2.797	2.825	2.790	3.476
*K*_*R*_	g-S/L	22.8	22.3	25.9	18.1	26.4	2.3	28.5	11.4
*K*_*I*_	g-S/L	5.2	3.1	5.3	3.9	5.3	0.9	5.3	3.4
${\mu}_{P}^{o}$	g-P/g-R·h	0.894	1.177	0.844	1.350	0.839	1.049	0.804	0.708
*K*_*P*_	g-S/L	45.1	5.3	42.4	0.7	42.2	18.9	40.3	54.2
*Y*_*SX*_	g-S/g-X	0.772	1.426	0.864	1.348	0.881	1.849	0.941	2.303
${\mu}_{E}^{o}$	-	2.280	1.904	2.208	2.197	2.135	0.187	2.113	1.162
*k*_*1*_	L/mol-OH·s	1.821	3.001	1.805	3.569	1.809	3.798	1.796	0.736
*k*_*2*_	·10^–5^ L/g-D·s	1.739	1.455	1.700	1.079	1.699	0.444	1.671	3.174
*R*^*2*^	–		0.922		0.915		0.925		0.907
*S*_*Rm*_	g-S/L	4.38	2.79	4.99	3.32	4.51	0.71	4.59	2.78
*μ*_*Rm*_	g-R/g-R·h	0.196	0.148	0.177	0.362	0.174	0.316	0.164	0.303
*S*_*Pm*_	g-S/L	4.72	2.22	4.75	1.35	4.75	0.89	4.76	3.25
*μ*_*Pm*_	g-P/g-R·h	0.034	0.171	0.035	0.628	0.035	0.018	0.035	0.016

*2AB* binary mixture of acetic and butyric acid, *3HC* ternary mixture of acids at high concentration; 3LC ternary mixture of acids at low concentration, *DFE* real dark fermentation effluent; *pro* prorated values; *fi:* fitted values; *S*_*Rm*_ substrate concentration for the maximal specific growth rate; *μ*_*Rm*_ maximal specific growth rate; *S*_*Pm*_ substrate concentration for the maximal specific production rate; *μ*_*Pm*_ maximal specific production rate.

In relation to growth kinetic, the maximum specific growth rates (${\mu}_{R}^{o}$) for combined VFAs increase, while the other two parameters (*K*_*R*_ and *K*_*I*_) decrease. These changes lead to growth-substrate curves with maximal values of *μ*_*Rm*_ that are higher than expected (data included in Table 3). Specially, in the experiments at low substrate concentrations. Moreover, these rates could also be achieved at lower substrate concentrations than expected (Table 3). Thus, faster growth can be obtained if the VFAs are combined, even at lower concentration than separate. As for the production kinetic, again the specific production rates increase but the saturation and inhibition constants decrease. As a result, the production-substrate curves show maximal values higher than expected in some cases but lower in others (Table 3). However, in all cases these rates are obtained at lower substrate concentration than expected. Again, the combination of VFAs could yield some benefits over using them in single mode.

The yield coefficient of substrate to biomass (*Y*_*SX*_) increase significantly for combined VFAs, indicating that more quantity of carbon source must be consumed for the bulk growth in these cases, probably because substrate is used for the productions of different metabolites (or a higher quantity of some of them, as PHA) that need more mass and energy consumption. As for the proportionality constant for biomass degradation (${\mu}_{E}^{o}$), it was found to be lower in mixtures of VFAs. This suggests that residual cellular biomass is less susceptible to degradation under these specific conditions. A potential explanation for this is that biomass has more survival possibilities when several carbon sources and several metabolic routes for growth are in action. Additionally, the direct rate constant (*k*_*1*_) is found to be significantly higher in VFA mixtures and the reverse constant (*k*_*2*_) is found to be lower, which demonstrates that the polymer obtained in these cases is also more stable than before.

#### Real dark fermentation effluents

In the experiment with real fermentation effluents **(**Fig. 3d) the observed kinetic patterns were similar to the previous ones (acid mixtures), and the fitting parameters show similar deviations from the prorated values than the VFA mixtures (Table 3). However, the production parameters deviate differently, that is, the specific production rate decreases but the saturation constant increases. This leads to a production-substrate curve with the maximal value lower than expected (Table 3), suggesting that this type of DFE is not as propitious as the synthetic VFA mixtures for PHA production. The reason may lie in the lack of some type of nutrient in the fermented algal substrate (low content of phosphorous) or the presence of some type of inhibitor (high content of phlorotannins). Moreover, the direct rate constant (*k*_*1*_) decreases but the reverse constant (*k*_*2*_) increases, in relation to the prorated values, just the opposite of what happened with mixtures. This indicates that the PHAs obtained from DFE are also less stable. Once again, showing the superior suitability of synthetic VFA mixtures over these DFE.

**Fig. 4:**
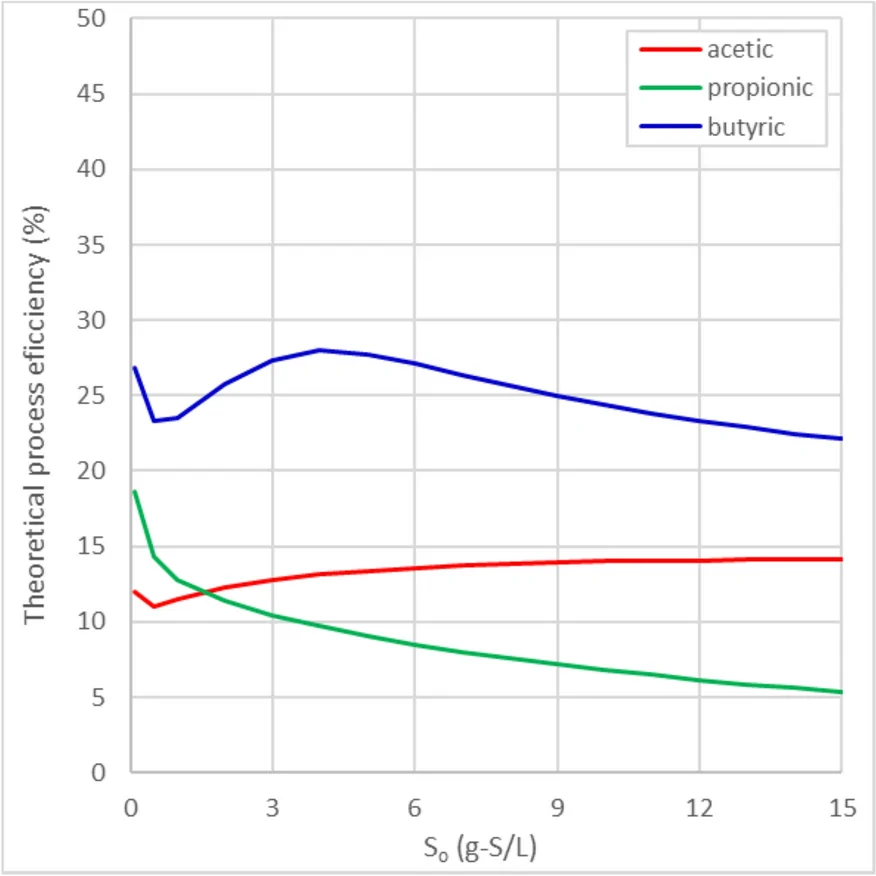
Theoretical process efficiency expressed in % (ratio of conversion of initial substrate mass to maximal polymeric product, 100 · g-P_max_/g-S_o_)

Apart from that, the general process efficiency, that is, the conversion ratio of initial substrate mass (g-S_o_/L) to polymeric product determined at the maximal P concentration (g-P_max_/L), was limited to approximately 2.4 % for the DFE experiments. This result coincides with the efficiency obtained for the single glucose experiments (3.1 %) and for the VFA mixture at low concentration (4.0 %). However, for the VFA mixtures at high concentration and for the single VFAs experiments, the obtained efficiencies were around four times higher, as has been mentioned before (2AB: 18.9 %; 3HC: 17.1 %; ACE: 15.7 %; PRO: 6.7 %; and BUT: 25.6 %). Furthermore, a wide set of simulations for the three single VFA (at initial substrate concentrations from 0 to 15 g-S/L) fails to improve significantly these results. A representation of the theoretical efficiencies can be seen in Fig. 4. Of course, the greater the substrate concentration supplied, the greater the product concentration obtained, but investing in increasing the VFA concentration of the DFE (water elimination) or in supplementing the natural effluents with pure VFAs, does not seem advisable with such low yields. However, the possibility that different real DFE present different matrices (nutrients or inhibitors) could drive to different set of model parameters and different results. This confirms the need of develop more robust kinetic models, in order to identify the precise window of operating conditions required to maximize the productivity of the industrial-scale processes.

## Conclusions

The fermentation process for the synthesis of polyhydroxyalkanoates (PHA) involving *C. necator* has revealed a critical dependency on pH dynamics, which simultaneously governs microbial growth and polymer degradation. Consequently, the development of a kinetic model that integrates these biological and chemical phenomena simultaneously has proven valid for qualitatively estimating the empirical observations. Moreover, the proposed mathematical framework offers sufficient flexibility to be applied to diverse volatile fatty acids, their mixtures or to real DFE, bearing in mind that the set of parameters differs in each case.

Experimental results substantiate that PHA accumulation is hindered under conditions of low substrate concentration, whereas the proliferation of the active biomass is subject to inhibition under elevated acid titers. Furthermore, the transition toward alkaline conditions (a direct consequence of acid consumption) functions as a driver for the chemical decomposition of the biopolymer. This comprehensive modeling approach, which synthesizes the biochemical and chemical aspects of PHA production, constitutes a fair and slightly complex optimization tool for bioplastic production from dark fermentation effluents, thereby facilitating the transition toward sustainable industrial processes and a circular economy.

## Symbols

DFE: Dark fermentation effluents
*k*_*1*_: Second order kinetic constant for the forward reaction of the alkaline depolymerization of PHA (L/mol-OH·s)
*k*_*2*_: Second order kinetic constant for the reverse reaction of the alkaline depolymerization of PHA (L/g-D·s)
*K*_*R*_: Monod saturation constant for the biomass growth, referred to its residual part R (g-S/L)
*K*_*P*_: Saturation constant for the production of PHAs by biomass, referred to its residual part R (g-S/L)
*K*_*I*_: Substrate inhibition constant for biomass growth and PHA production, referred to its residual part R (g-S/L)
*OH*: Concentration of the hydroxyl anion in the fermentative medium (mol-OH/L)
*P*: Overall concentration of product (PHAs) in the fermentative medium, usually inside the cells (g-P/L)
PHA: Polyhydroxyalkanoate (PHAs in plural)
*R*: Concentration of residual biomass, i.e. the part of biomass in dry weight that is not P product (g-DW/L)
*R*^*2*^: Non-linear determination coefficient (dimensionless. between 0 and 1)
*S*: Total substrate concentration in the fermentative medium, in this case VFAs (g-S/L)
*t*: Process time (h)
VFA: Volatile fatty acid (VFAs in plural)
*X*: Total concentration of microbial biomass in dry weight (g-DW/L)
*Y*_*SX*_: Yield coefficient substrate to biomass (g-S/g-X). Amount of substrate consumed per unit of total biomass formed

### Greek letters

*μ*_*D*_: Specific decomposition rate of the product P, referred to the residual part of biomass R (g-P/g-P·h)
*μ*_*E*_: Specific degradation rate of biomass, referred to its residual part R (g-R/g-R·h)
*μº*_*E*_: Proportionality constant between *μ*_*E*_ and *μ*_*D*_, (dimensionless)
*μ*_*P*_: Specific production rate of PHAs by biomass, referred to its residual part R (g-P/g-R·h)
*μ*_*R*_: Specific growth rate of biomass, referred to its residual part R (g-R/g-R·h)

### Subscripts

_o_: The initial value of any variable (for example *S*_*o*_)
_m_: The maximal value of any parameter (for example *μ*_*Rm*_) or the value of any variable to produce this maximal value (for example *S*_*Rm*_).

### Superscripts

^o^: The reference value of any parameter (for example. ${\mu}_{R}^{o}$)

## Data Availability

No datasets were generated or analysed during the current study.
